# Cannabis-Induced Catatonia Complicated by Rhabdomyolysis, Acute Kidney Injury, and Sympathetic Overactivity: A Case Report

**DOI:** 10.7759/cureus.97507

**Published:** 2025-11-22

**Authors:** Sidharth Saira, Himanshi Singh

**Affiliations:** 1 General Medicine, Chelsea and Westminster Hospital, London, GBR; 2 Acute Medicine, Chelsea and Westminster Hospital, London, GBR

**Keywords:** acute kidney injury, benzodiazepines, cannabis, catatonia, psychosis, rhabdomyolysis, sympathetic overactivity

## Abstract

Catatonia is a severe neuropsychiatric syndrome characterized by motor, behavioral, and autonomic disturbances. It can result in life-threatening complications such as rhabdomyolysis, venous thromboembolism, acute kidney injury, and autonomic instability. Substance use, particularly cannabis, is increasingly recognized as a precipitant, especially in vulnerable individuals with prior psychotic episodes. We report the case of a 21-year-old male patient with a history of cannabis-induced psychosis who presented with mutism, social withdrawal, psychomotor retardation, and poor oral intake following non-compliance with depot antipsychotics and recent cannabis use.

On examination, he exhibited thought blocking, waxy flexibility, negativism, and stupor. Investigations revealed markedly elevated creatine kinase (CK), indicative of rhabdomyolysis, mild renal impairment(raised creatinine), and urine toxicology positive for tetrahydrocannabinol. Other laboratory investigations, including hematologic, hepatic, thyroid, and metabolic panels, along with CT head and chest X-ray, were unremarkable, helping to rule out alternative organic causes. A comprehensive evaluation ruled out primary psychiatric disorders, antipsychotic-induced catatonia, and organic causes. Psychotic symptoms emerged concurrently with catatonic features, with recent cannabis use preceding symptom onset and a prolonged symptom-free interval prior to presentation, supporting a diagnosis of cannabis-induced catatonia in a patient with a history of cannabis-related psychosis. The patient was managed with intravenous fluids, lorazepam, aripiprazole, and clonazepam. Close cardiac monitoring was implemented for recurrent tachycardia. Over the hospital stay, psychomotor function and catatonic features gradually improved, with normalization of CK and renal function. Residual mild psychomotor slowing and psychotic ideation persisted.

This case report presents an instance of catatonia associated with cannabis use in a young adult with a prior history of cannabis-related psychosis. It explores the clinical reasoning used to distinguish this presentation from other potential causes, including mood disorders, antipsychotic-related effects, and medical or organic conditions, and discusses evidence-based management approaches aimed at minimizing medical complications and optimizing patient outcomes. It also highlights the need for patient education on cannabis-related neuropsychiatric risks and routine substance use screening in psychiatric populations.

## Introduction

Catatonia is a complex psychomotor syndrome characterized by abnormalities in movement, behavior, and responsiveness, occurring in approximately 10% of acutely unwell psychiatric patients. It may be observed in psychiatric conditions, including mood disorders, schizophrenia, drug-induced psychosis, and neurodevelopmental disorders, as well as in medical and neurological illnesses. Clinical presentations range from profound unresponsiveness to excessive motor activity, often leading to under-recognition [1,2]. 

The pathophysiology involves disruptions in gamma-aminobutyric acid (GABA) and glutamate neurotransmission, contributing to both motor abnormalities and autonomic dysregulation [3]. Neuroimaging studies demonstrating decreased GABA receptor binding, along with the often rapid therapeutic response to benzodiazepines, support a model of GABAergic hypofunction with secondary dysregulation of excitatory pathways such as glutamate. 

Catatonia presents in distinct subtypes with clinical implications: akinetic (immobility, mutism, fixed gaze), excited (excessive purposeless activity, agitation), and malignant (autonomic instability, hyperthermia, rapid deterioration) [3]. Malignant catatonia shares clinical features with neuroleptic malignant syndrome (NMS); however, early catatonic symptoms and a typically robust response to benzodiazepines help differentiate between the two conditions [4,5]. 

Substance use, particularly cannabis and synthetic cannabinoids, is increasingly recognized as a trigger for catatonia [6,7]. Cannabis affects GABAergic neurotransmission through multiple mechanisms. Activation of presynaptic receptors by cannabinoids such as tetrahydrocannabinol (THC) inhibits the release of GABA from inhibitory interneurons, resulting in reduced inhibitory synaptic activity [8]. Conversely, certain cannabinoid compounds may enhance postsynaptic GABA receptor currents, acting as positive allosteric modulators under specific conditions. Overall, cannabis exerts a bidirectional modulatory effect on GABA signaling, attenuating presynaptic inhibition while potentially augmenting postsynaptic GABAergic responses [9]. 

Early identification is critical, as untreated catatonia can result in severe complications, including malignant catatonia due to autonomic instability, prolonged immobility leading to rhabdomyolysis and venous thromboembolism, dehydration, cardiac arrhythmias, and acute kidney injury. Therefore, prompt recognition and management are essential to prevent these potentially life-threatening outcomes [10].

## Case presentation

The patient was a 21-year-old male with a documented history of cannabis-induced psychosis previously treated with depot paliperidone, though non-compliant with follow-up and medication after discharge. He lived with his family, who reported ongoing cannabis use of unquantified frequency, occasional nicotine vaping, and no other substance use. There was no other relevant past medical or drug history, and no family history of psychotic disorders. Prior to the current presentation, he had been hospitalized one year earlier for acute psychosis, which resolved with antipsychotic treatment. Notably, he exhibited no catatonic features during that admission and remained asymptomatic during travel abroad nine months later.

He was brought to the emergency department by ambulance after three to four days of mutism, markedly reduced oral intake, insomnia, and social withdrawal. Preceding admission, his mother had noted a decline in communication, psychomotor slowing, and ongoing cannabis use. She also reported episodes of confusion, talking to himself, and the patient mentioning hearing voices before becoming largely unresponsive. No recent illness, stressors, or medication changes were identified.

On arrival, he was alert but minimally responsive, displaying thought blocking, monotone speech, and a perplexed affect. He was unable to account for his symptoms and repeatedly stated that “something was wrong.” His mother confirmed recent cannabis use following a period of earlier abstinence, although the amount and frequency were unknown.

Vital signs showed he was afebrile with blood pressure 154/93 mmHg, pulse 122 beats/minute (bpm), respiratory rate 18 breaths/minute, and saturation of peripheral oxygen (SpO₂) 97% on room air. He was appropriately dressed, with a blunt affect and poor eye contact. Neurological and systemic examinations were unremarkable apart from psychomotor retardation, mild waxy flexibility, negativism, and periods of decreased responsiveness. 

Laboratory testing revealed a markedly elevated creatine kinase (CK) (25,016 U/L) consistent with rhabdomyolysis, along with mildly increased serum creatinine (Table 1). Electrolytes were normal, and urinalysis was unremarkable aside from a positive toxicology screen for THC. CT head and chest X-ray showed no acute abnormalities. ECG demonstrated sinus tachycardia at 122 bpm with a QTc of 422 ms.

**Table 1:** Blood investigations on admission Elevated values are shown in bold

Parameter	Patient Value	Reference Range	Units
Haemoglobin	142	130-180	g/L
White blood cells count	7.5	3.6-11	×10⁹/L
Platelets count	188	140-400	×10⁹/L
Urea level	4.5	2.5-7.8	mmol/L
Creatinine	109	59 – 104	µmol/L
Sodium level	141	133 – 146	mmol/L
Potassium level	4.2	3.5 – 5.3	mmol/L
Alanine aminotransferase	73	5 – 40	IU/L
Alkaline phosphatase	108	25 – 115	IU/L
Albumin level	38	38 – 50	g/L
Total bilirubin	25	0 – 17	µmol/L
C-reactive protein	2.4	<5	mg/L
Thyroid stimulating hormone	3.2	0.4-4.0	mU/L
Adjusted calcium	2.21	2.2–2.6	mmol/L
Magnesium	0.91	0.75–1.0	mmol/L
Phosphate	0.82	0.8–1.5	mmol/L
Creatine kinase	25016	40-320	U/L

He was admitted and commenced on one-to-one observation. Intravenous fluids were initiated for rhabdomyolysis. Lorazepam 1-2 mg every 12 hours was started for catatonic features. On admission, his estimated Bush-Francis Catatonia Rating Scale (BFCRS) score was 22/69. Within 48 hours, improvements were observed, with increased responsiveness and improved oral intake, corresponding to an estimated BFCRS reduction to approximately 15/69.

On day three, oral aripiprazole 10 mg daily was introduced, later increased to 20 mg daily over the week. Clonazepam 1 mg three times daily was added on day four for intermittent agitation. Tachycardia was monitored and did not progress to further arrhythmia.

By day seven, CK levels had normalised, renal function improved, and there was a marked reduction in catatonic symptoms, with an estimated BFCRS score of approximately 5/69. The patient became more conversational and compliant with care. Electroconvulsive therapy (ECT) was not required, as symptoms responded to benzodiazepines and supportive measures.

## Discussion

A range of differential diagnoses was considered in this case. Primary psychotic disorders, including schizophrenia, were explored, given the patient’s psychiatric history; however, there was no evidence of persistent psychotic symptoms independent of substance use. The temporal relationship between recent cannabis use, medication non-adherence, and the onset of catatonic features suggested a possible substance-related etiology. Mood disorders were considered less likely, as there were no preceding or concurrent affective symptoms. Organic causes such as infection, metabolic derangement, or structural brain abnormalities were excluded through clinical assessment, laboratory testing, and neuroimaging. Neuroleptic malignant syndrome was also considered, but was deemed less probable due to the absence of fever or recent antipsychotic exposure. Antipsychotic-induced catatonia was unlikely, as the patient had not received depot medication recently.

Taken together, the pattern of findings was most consistent with cannabis-associated catatonia, while acknowledging that overlapping vulnerabilities, including prior psychotic episodes, may have contributed to presentation. Antipsychotic non-compliance likely increased vulnerability to relapse. Prolonged immobility led to clinical complications, including rhabdomyolysis (CK 25,016 U/L), dehydration, and early acute kidney injury, while autonomic instability manifested as recurrent tachycardia, necessitating close monitoring and supportive care.

Diagnosing catatonia in an acute setting is challenging; however, early recognition and prompt management are critical to prevent life-threatening complications. As per the Diagnostic and Statistical Manual of Mental Disorders, Fifth Edition, Text Revision (DSM-5-TR) [11], catatonia is diagnosed when at least three of 12 characteristic symptoms listed in Table 2 are present.

**Table 2:** Diagnostic criteria as per DSM-5-TR. DSM-5-TR: The Diagnostic and Statistical Manual of Mental Disorders, Fifth Edition, Text Revision [11]

Symptom	Meaning
Stupor	No psycho-motor activity;not actively relating to environment
Catalepsy	Passive induction of a posture held against gravity
Waxy flexibility	Slight, even resistance to positioning by examiner
Mutism	No , or very little, verbal response [exclude if known aphasia]
Negativism	Opposition or no response to instructions or external stimuli
Posturing	Spontaneous and active maintenance of a posture against gravity
Mannerism	Odd, circumstantial caricature of normal actions
Stereotypy	Repetitive, abnormally frequent, non-goal- directed movements
Agitation	Which is not influenced by external stimuli
Grimacing	Distorted , Distressed look
Echolalia	Mimicking another’s speech
Echopraxia	Mimicking another’s movements

A comprehensive evaluation is essential to exclude underlying neurological or medical causes. Laboratory investigations should include hematologic, renal, hepatic, thyroid, and metabolic panels, CK levels, and urinalysis to identify comorbidities and complications. Continuous monitoring of vital signs is crucial, as fever, hypertension, and elevated CK may indicate malignant catatonia or neuroleptic malignant syndrome. A thorough medication history should also be obtained, given the association of catatonia with antipsychotic use and abrupt benzodiazepine withdrawal. Despite limitations imposed by immobility, selected elements of the neurological examination, such as cranial reflexes, tone, and deep tendon reflexes, can typically be assessed. EEG and MRI may also be considered to exclude structural or functional abnormalities.

Benzodiazepines, particularly lorazepam, remain the first-line therapy for acute catatonia due to their ability to enhance GABA receptor activity, which helps normalize inhibitory neurotransmission and reduce motor and behavioral symptoms of catatonia [12]. They are generally safe and rapidly effective, with many patients showing improvement within hours to days of initiation.

ECT is reserved for severe, refractory, or life-threatening catatonia or when patients do not respond adequately to benzodiazepines. It involves the induction of a brief, controlled seizure under anesthesia, which is thought to reset dysfunctional neural circuits involved in motor, affective, and cognitive regulation. Its use requires careful consideration of clinical risks, availability, legal requirements, and patient consent, and is guided by multidisciplinary psychiatric and medical teams [13,14]. In this case, ECT was not required, as the patient responded effectively to benzodiazepine therapy combined with supportive care. Combining benzodiazepines with ECT may provide synergistic benefits [15], though large-scale, controlled studies are lacking. Antipsychotics should be introduced cautiously once catatonia begins to resolve, while autonomic instability requires vigilant monitoring to prevent cardiovascular collapse.

**Figure 1:**
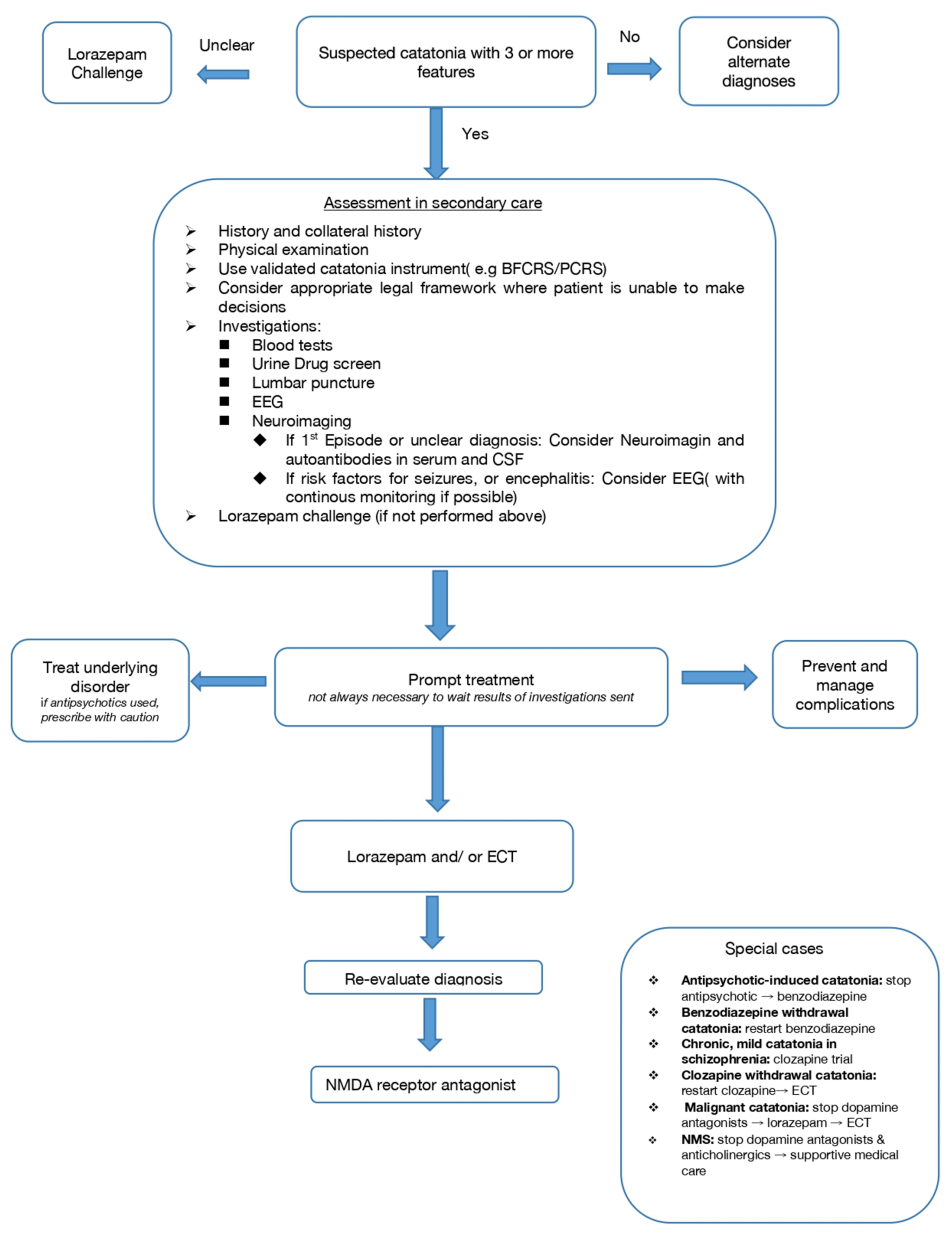
Summary of treatment algorithm for catatonia ASD: autism spectrum disorder; BFCRS: Bush-Francis Catatonia Rating Scale; CSF: cerebrospinal fluid; ECT: electroconvulsive therapy; EEG: electroencephalography; NMDA: N-methyl-D-aspartate; NMS: neuroleptic malignant syndrome; PCRS: Pediatric Catatonia Rating Scale Image adapted from Rogers et al., 2021 [16]; published under CC BY 4.0, Attribution 4.0 International Deed

In 2023, the British Association for Psychopharmacology produced a consensus guideline for the management of catatonia, which is summarised in Figure 1 [16].

Although the patient’s catatonia was temporally related to recent cannabis use, individuals with cannabis-induced psychosis carry an increased risk of developing subsequent schizophrenia-spectrum disorders, underscoring the need for ongoing vigilance [17]. Regular psychiatric follow-up, monitoring for early psychotic symptoms, and education regarding substance use are therefore essential to reduce the likelihood of progression to persistent psychotic illness.

## Conclusions

This report illustrates the severe medical and psychiatric complications that can arise from catatonia in the context of cannabis-induced psychosis. The patient’s acute presentation, with mutism, psychomotor retardation, rhabdomyolysis, and early renal impairment, highlights the importance of early recognition and comprehensive evaluation to prevent life-threatening sequelae. The diagnosis of cannabis-induced catatonia was supported by the temporal relationship between recent cannabis use, prior history of cannabis-induced psychosis, non-compliance with depot antipsychotics, and the exclusion of alternative causes, including schizophrenia, mood disorders, neuroleptic malignant syndrome, and organic medical conditions. History from the patient, along with good collateral information from the family, corroborated laboratory findings and imaging, further reinforcing this differentiation. Management with benzodiazepines (lorazepam), supportive care including IV fluids, and careful reintroduction of antipsychotics led to marked improvement in psychomotor function and catatonic features. ECT was not required, demonstrating that prompt pharmacological and supportive interventions can be sufficient in many cases.

This case also underscores the broader public health implications, emphasizing the need for early screening for cannabis use in psychiatric populations, patient education on the neuropsychiatric risks of cannabis, and close monitoring for relapse in individuals with a history of substance-induced psychosis. Regular psychiatric follow-up and monitoring for early psychotic symptoms are essential to reduce the risk of progression to persistent psychotic illness. By contributing to the growing literature linking cannabis use to catatonia, this case further highlights the importance of proactive, multidisciplinary approaches to optimize patient outcomes and prevent severe neuropsychiatric complications
